# Successfully treated congenital cystic adenomatoid malformation by open fetal surgery: A care-compliant case report of a 5-year follow-up and review of the literature

**DOI:** 10.1097/MD.0000000000005865

**Published:** 2017-01-13

**Authors:** Dazhi Fan, Shuzhen Wu, Rui Wang, Yi Huang, Yao Fu, Wen Ai, Meng Zeng, Xiaoling Guo, Zhengping Liu

**Affiliations:** a Foshan Fetal Treatment Center; b Department of Obstetrics, Southern Medical University Affiliated Maternal & Child Health Hospital of Foshan, Foshan, Guangdong; c Department of Epidemiology and Biostatistics, School of Public Health, Anhui Medical University, Hefei, Anhui, China.

**Keywords:** case report, congenital cystic adenomatoid malformation, follow-up, open fetal surgery

## Abstract

**Background:**

Congenital cystic adenomatoid malformation (CCAM) is a rare hamartomatous cystic lesion. Open fetal surgery currently provides a potential therapeutic option for management of a fetus with CCAM diagnosis.

**Case Summary:**

A 22-year-old G2P0 woman presented at   weeks’ gestation for evaluation of a fetus with a left lung lesion and diagnosed as CCAM at   weeks’ gestation. Open fetal surgery was performed to resection the lesion at   weeks’ gestation under deep maternal general anesthesia. The mother presented at   weeks after open fetal surgery with preterm premature rupture of membranes (PPROM) and underwent cesarean delivery at   weeks’ gestation. A vigorous woman infant of 1955 g, with good Apgar score, was delivered. At 1 month, 4 years, and present, 5 years after birth, she has continued to do well without any obvious deficit and both respiration and circulation were well maintained.

**Conclusion:**

We present one case of CCAM which was cured by open fetal surgery and continued to do well at follow-up of 5 years. The success of treatment provided preliminary experience for further carrying out such interventions in China.

## Abstract 2

Supplemental Digital Content is available in the text

## 1 Introduction

Congenital cystic adenomatoid malformation (CCAM), also called congenital pulmonary adenomatoid malformation (CPAM), is a rare hamartomatous cystic lesion, firstly described by Ch’In and Tang in 1949,^[1]^ and classified into 3 subtypes in 1977,^[2]^ and expanded into 5 types with a new name as CPAM by Stocker in 2002.^[3]^ In Canada, the condition occurs in 1 in 25,000 to 35,000 births.^[4]^ Our previous study noted that the occurrence is approximately 3.34:10,000 in China,^[5]^ nearly 10 times higher than the level reported in Canada. Prenatal intervention of CCAMs was determined by the size and classification of the lesion as well as the presence of fetal hydrops. Open fetal surgery currently provides a potential therapeutic option for management of the fetus with CCAM diagnosis and massive nonimmune hydrops. We present one case of CCAM, which was cured by open fetal surgery and continued to do well after a follow-up of 5 years. To our knowledge, this is the first report in China.

## 2 Case presentation

This case presentation has been consented by the family and approved by the ethics committee of South Medical University Affiliated Maternal & Child Health Hospital of Foshan.

A 22-year-old G2P0 woman presented at  
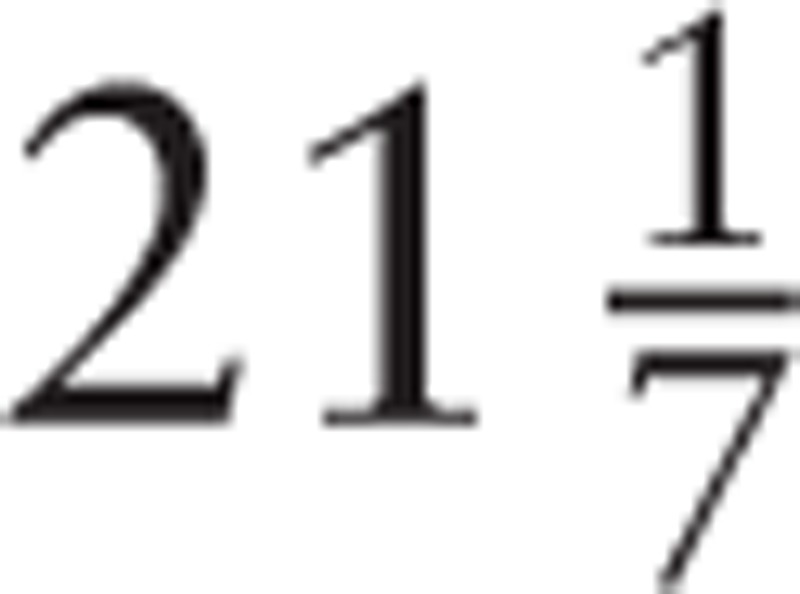
 weeks’ gestation for evaluation of a fetus with a left lung lesion. A detailed ultrasound examination considered pulmonary sequestration, but pulmonary cystadenoma could not be totally excluded. Serial ultrasonographic monitoring demonstrated an increasing size of the lesion of the left lung and the heart was shifted to the right. The ultrasound examination revealed a 5.6 cm × 5.0 cm × 5.0 cm mass at  
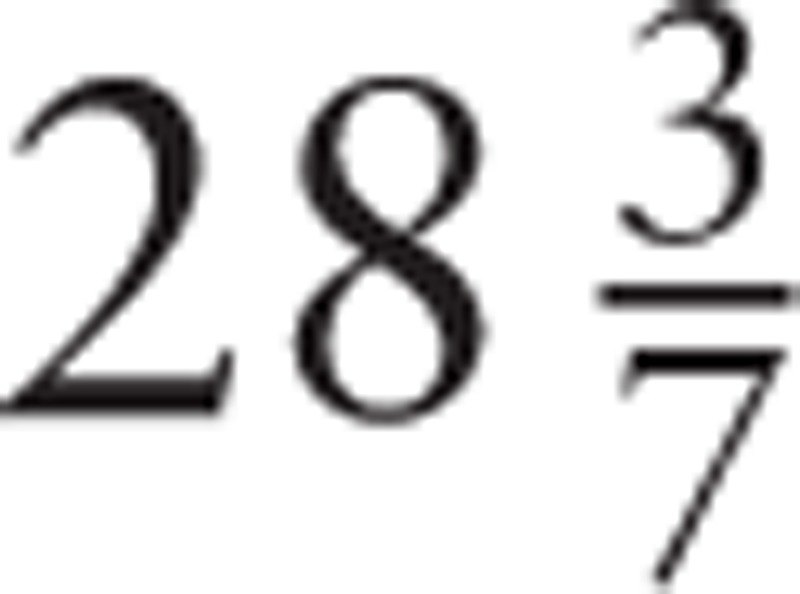
 weeks’ gestation (Fig. 1). The presentation was believed to be consistent with a CCAM. The cystic adenomatoid malformation volume ratio (CVR) was 2.8. Amniocentesis confirmed normal karyotype and no other anatomic abnormalities were present on detailed ultrasonographic survey.

**Figure:**
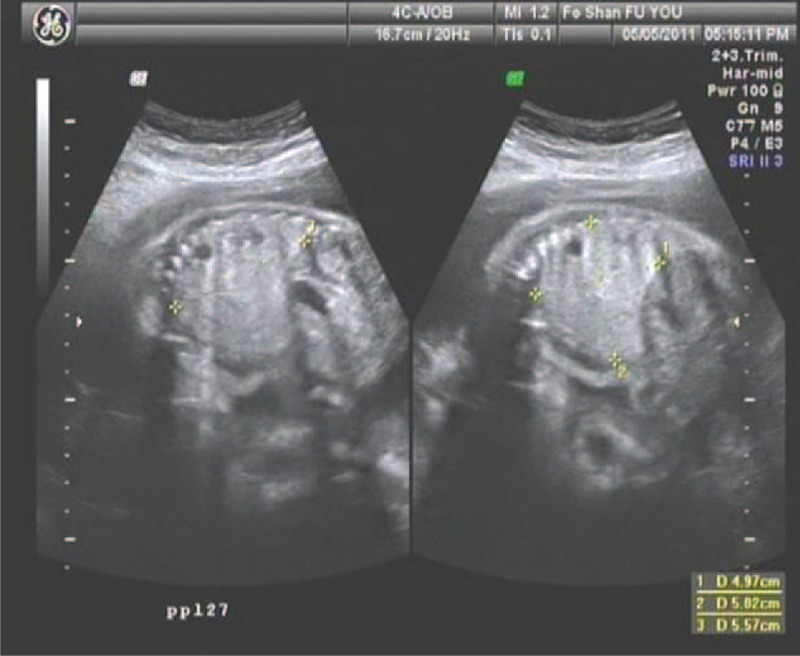


**Figure 2:**
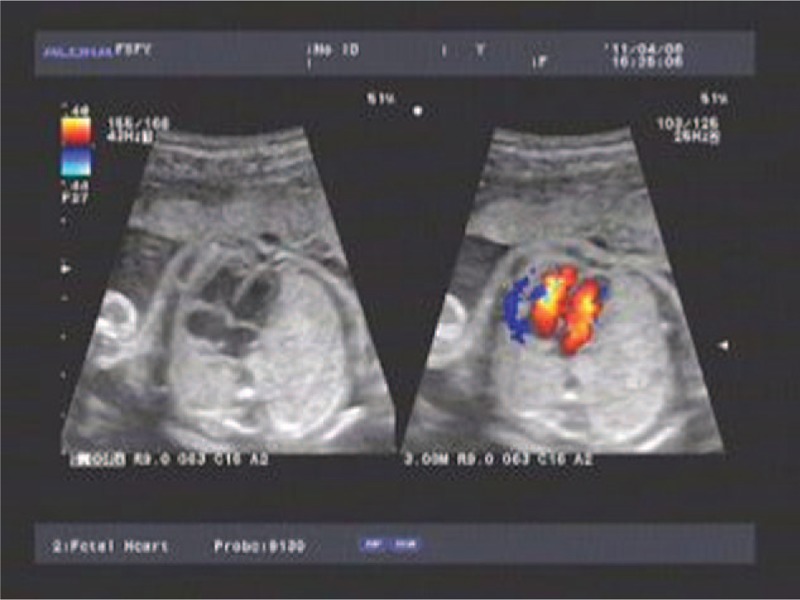
Sonographic measurement of the heart function.

Nonimmune hydrops were not found before operation, and the heart function was well preoperatively (Fig. 2). Given the continued growth of the lung mass, the concern for the progression of hydrops and fetal demise, and the significant pulmonary hypoplasia believed incompatible with survival secondary to mass effect, multidisciplinary counseling was performed regarding open fetal surgery. After full evaluation and counseling by the fetal surgery team, the patient and her family wished to proceed with intrauterine surgery. Informed consent was obtained in accordance with the surgery protocol, and the open fetal surgery was approved by the ethics committee of South Medical University Affiliated Maternal & Child Health Hospital of Foshan.

A maternal hysterotomy, left fetal thoracotomy and CCAM resection were performed at  
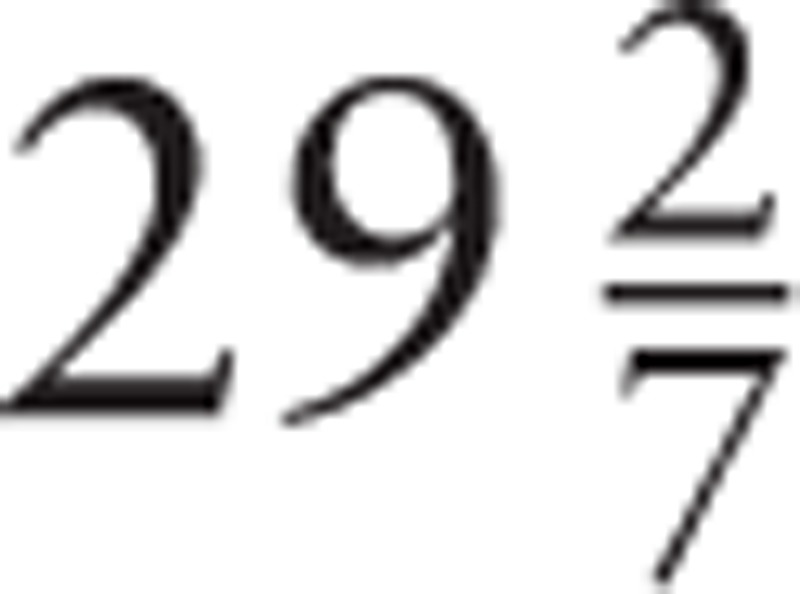
 weeks’ gestation (May 15, 2011) under deep maternal general anesthesia.^[6]^ The open fetal surgery techniques were performed as described below.

Indomethacin and antibiotics were given preoperatively, and ritodrine hydrochloride provided the necessary uterine relaxation. Sterile intraoperative sonography delineated both the fetal and placental position. The uterine incision (about 10 cm) was made at the edge of the placenta.^[7]^ The left fetal hand was delivered, intravenous access obtained, and a fetal pulse oximeter was placed. A left fetal thoracoabdominal incision was made based in the fifth intercostal space. Continuous fetal echocardiography assessed ongoing cardiac function and directed resuscitation requirements. After the thoracotomy, the mass was slowly delivered from the chest cavity and the CCAM resection was initiated. The fetal chest wall was closed and the fetus returned to the uterine cavity. An intrauterine dose of dexamethasone (10 mg) and ampicillin (4 g) were administered after the amniotic cavity was filled with warmed (37 °C) lactated Ringer solution. And the uterine and abdominal incisions were closed in layers. The mother tolerated the procedure well and was given atosiban and ritodrine hydrochloride after the surgery. Continuous fetal echocardiography confirmed recovery of fetal heart function after the intraoperative fetal resuscitation.

**Figure 3:**
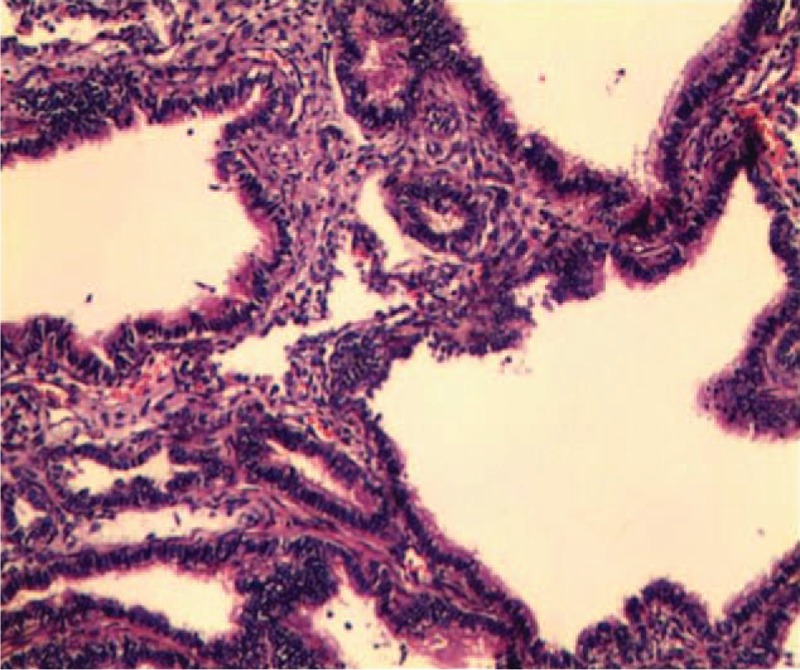
Histopathological study of the resected lung specimen revealed multiple cysts.

**Figure 4:**
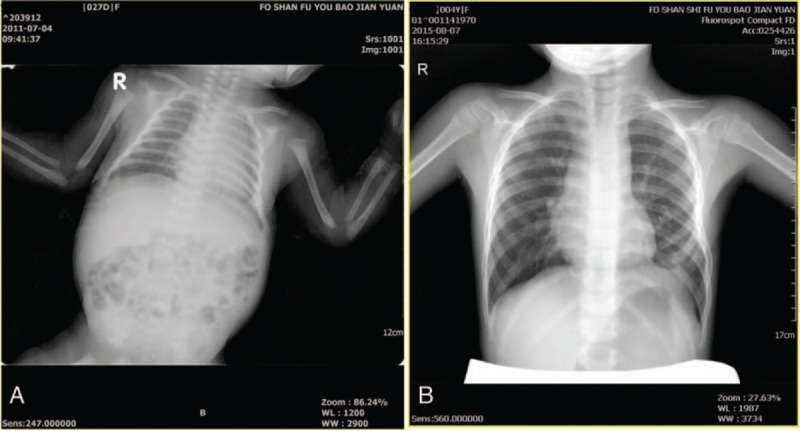
Chest radiographs. (A) At 25 days after birth. (B) At 4 years after birth.

Postoperatively, the mother was treated with intravenous magnesium sulfate, indocin, and transitioned to nifedipine for the remainder of the pregnancy to maintain uterine quiescence. The pathology from the fetal excision demonstrated pulmonary mass hyperplasia consistent with CCAM-like changes (Fig. 3). The mother presented at  
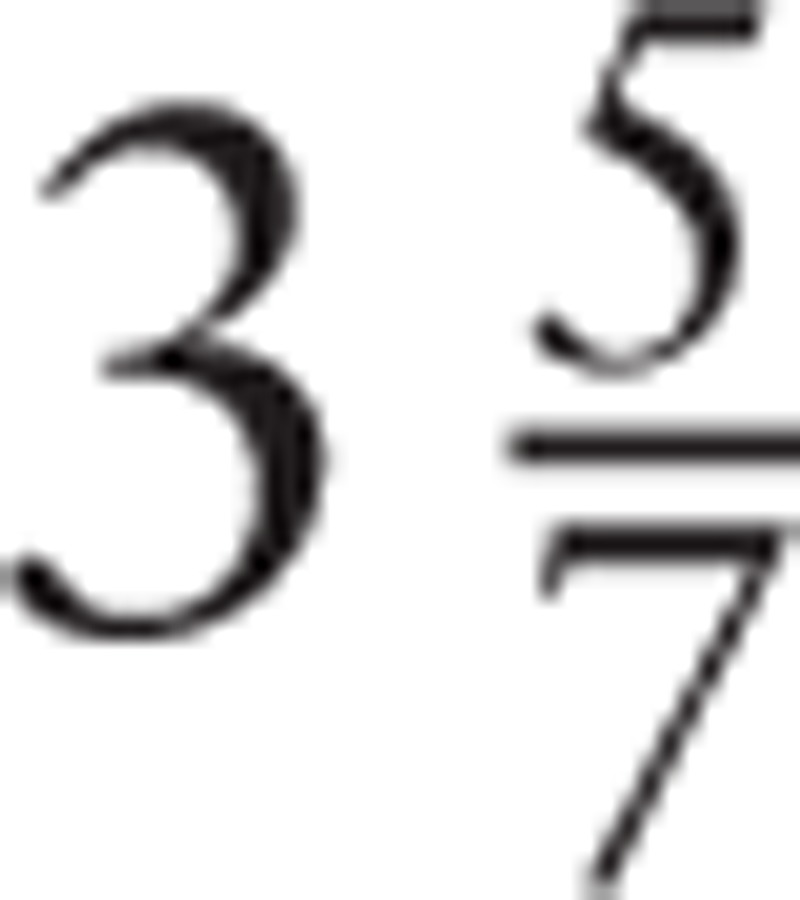
 weeks after open fetal surgery with preterm premature rupture of membranes (PPROM) and underwent cesarean delivery at  
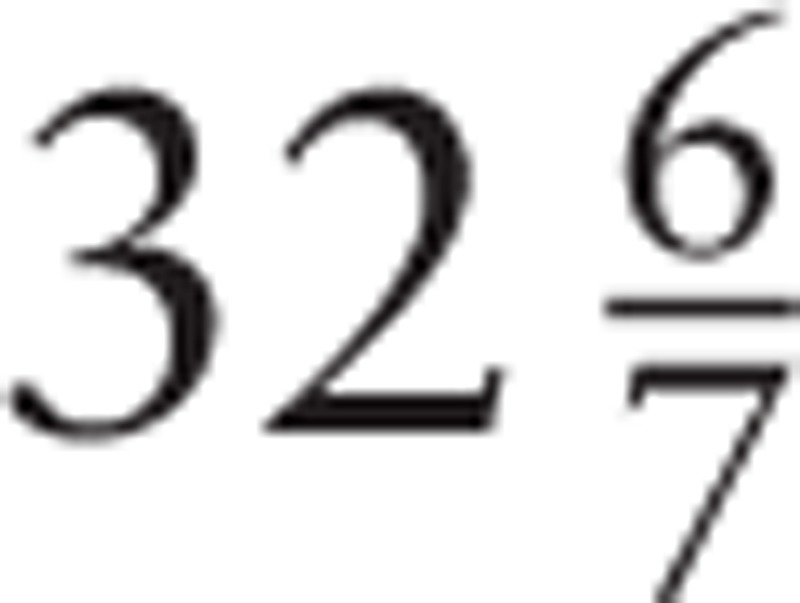
 weeks’ gestation. A vigorous woman infant of 1955 g (50th centile), with good Apgar score, was delivered. The baby's thoracoabdominal incision was well healed at birth without any evidence of fluid leakage. At 1 month, 4 years, and present, 5 years after birth, she has continued to do well without any obvious deficit and both respiration and circulation were well maintained (Fig. 4A, B).

**Figure 5:**
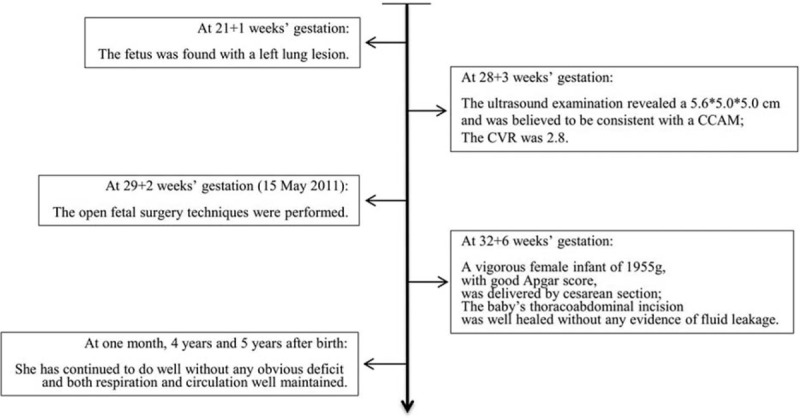
Progression of the patient's condition and accompanying interventions.

Progression of the patient's condition and accompanying interventions are illustrated in a flowchart (Fig. 5). The surgery and follow-up are displayed in supplemental video.

## 3 Discussion

CCAM is a rare fetal pulmonary lesion, it can be diagnosed in antenatal period by ultrasonography. It is the most frequently observed lung lesion (25%) among all subtypes of congenital pulmonary malformations, and most cases are found in neonates and infants while pulmonary malformations are infrequent in adulthood.^[8]^ Lung lesions are diagnosed nowadays mainly by prenatal ultrasound imaging, but magnetic resonance imaging can be very useful in the differential diagnosis of bronchogenic cysts, bronchial atresia, or multilobe involvement.

Almost 100% of lung cysts could be detected at the routine 18 to 20-week scan.^[9]^ It is extremely variable in the clinical spectrum and natural history of CCAMs, especially with respect to lesion growth during the canalicular period (17–29 weeks). It has been reported that without nonimmune hydrops fetalis, lung masses can be spontaneously disappear in the third pregnancy.^[10]^ However, the development of nonimmune hydrops fetalis portends 100% mortality rate in the presence of CCAM.^[11]^ Although spontaneous resolution of nonimmune hydrops have been reported, resolution of hydrops in association with fetal lung lesions is exceedingly rare.^[12]^

Without prenatal intervention, the presence of bilateral disease and hydrops fetalis are indicators of poor outcome,^[13]^ whereas early detection, polyhydramnios and mediastinal shift are not poor prognostic signs in the fetus with a CCAM.^[14,15]^ It will present significant cardiorespiratory compromise at birth in some postnatal patients. However, it can also be asymptomatic. When the other symptoms, such as recurrent respiratory infections, appear, it may be incidentally diagnosed in postnatal life when a chest computed tomography (CT) or X-ray is performed.

With advances in antenatal ultrasonography, CCAM has been increasingly diagnosed. Various ultrasonography features, including the size and type, presence of mediastinal shift and hydropic change, have been associated with a poor prognosis. Up till now, the exact mechanisms of the congenital disease remain unknown, although research found the lesion coexists with recombinant chromosome 18,^[16]^ and this pathology of karyotype could not be ruled out by certain.

Although postnatal intervention is possible, with increasing in utero diagnosis with ultrasound, the condition has been treated during pregnancy (prenatal intervention) with the aim of decreased disability and morbidity for the neonate and infant. Normal karyotype and without other detected anomalies are the major selection criteria for prenatal intervention.

There have been several interventions for the treatment of the CCAM. The interventions mainly include amniocentesis treatment,^[17]^ steroid therapy,^[12,18–20]^ thoracoamniotic shunts,^[21]^ laser ablation,^[22]^ and surgical treatments.^[23,24]^

Recently, several institutions^[12,18–20]^ have reported that steroid is an effective treatment for high-risk fetal lung masses. Although the specific mechanism is not clear, these results indicate that steroid therapy may be beneficial in certain cases. In addition, thoracoamniotic shunts may also be effective in secondary hydrops fetus with a large macrocysts. If the fetuses with hydrops, the thoracoamniotic shunt should be regarded as a kind of good treatment option to prevent the accumulation of liquid.^[21]^ Nevertheless, with thoracoamniotic shunts, there is the risk of premature labor, displacement of the shunt, or sepsis.^[25]^ For percutaneous laser ablation, it is still a limited experience to treat multicystic lesions. Although tumor size decreased, the hydrops worsened and fetal death occurred.^[22]^ Moreover, other study in Korean population found that percutaneous injection of OK-432, a sclerosing agent, is also a good choice to treat these fetuses.^[26]^

If there is no good response to other conservative treatments, open fetal surgery is also an effective treatment for fetuses with significantly hydrops and solid lung mass. There is no strict indication to undertake this intervention, as the preoperative cardial function is normal and there is no non-immune hydrops. The first open fetal surgery has been successful in salvaging a fetus with CCAM.^[27]^ Subsequently, the technique is gradually being reported to successful removal the lesion.^[23,28,29]^ We have previously reported 1 case of CCAM cured by open fetal surgery in 2011,^[30]^ and she is continued to do well follow-up 5 years. The ex-utero intrapartum treatment (EXIT) can be also considered if the fetuses with large lung masses at the end of gestation.^[31]^ The surgery technique can guarantee the placental gas exchange in the process of operation. Previous studies,^[24,32,33]^ including our team, have reported that it is also a very nice choice for those large lung masses fetuses.

Postnatal intervention is dictated by clinical status at birth. In asymptomatic cases, postnatal investigation consists of plain radiographic evaluation on the first day after delivery, and a chest CT scan within 1 month of birth.^[14]^ Symptomatic lesions require urgent radiological evaluation with chest radiography, followed by surgical excision. The management of the symptomatic lesions carries a higher morbidity.^[34]^ Although a series of studies indicate that it is performed safely on patients who were asymptomatic at birth,^[35]^ surgical excision of these is still controversial, with some centers opting for conservative management.^[36,37]^

Fetal surgical techniques, a potential therapeutic option, have been previously described 2 decades ago^[38]^ and survived tens of hydropic fetuses.^[39]^ In addition to CCAM, fetal surgery may benefit other congenital abnormalities, like as myelomeningocele, congenital diaphragmatic hernia, sacrococcygeal teratoma, and fetal airway obstruction. However, a similar operation has not been reported in China or other developing areas. To our knowledge, this is the first report that a fetus with CCAM was cured by open fetal surgery and continued to do well follow-up 5 years in China.

Multidisciplinary counseling and cooperation is the foundation of successful operation. As a comprehensive Children and Maternal hospital, we were able to assemble a fetal surgery team with active participation of ethicist, obstetrician, paediatric surgeon, obstetric anesthetist, neonatologist, and pediatrician. Meanwhile, patient safety and quality of care must be at the forefront of any institution's effort to offer fetal surgery. One limitation of our study is only 1 case is continued to do well at follow-up of 5 years. The applicability of the intervention and side-effect need large-sample and long-term follow-up.

In conclusion, we present 1 case of CCAM that was cured and continued to do well follow-up 5 years by open fetal surgery in China. The success of treatment provided preliminary experience for further carrying out such interventions.

## Acknowledgments

The author(s) thank the patient and her family for participation in the study. Thanks to Zhanhui Lu, in their department, helps them to show the ultrasound image of the hydrops and heart before operation.

## Supplementary Material

Supplemental Digital Content
